# Nephritis in Relation to the Recent Epidemic of Influenza

**Published:** 1919

**Authors:** 


### *Nephritis in Relation to the Recent Epidemic of Influenza.*

The Results of an Investigation of 22 Cases. By C. P. Symonds, R. A. M. C. *The Lancet*, November 16, 1918.

No patient gave a history of previous nephritis or of scarlet fever. The average age was 25.3 years. The average physique in the series was good.

In only one case of the series was marked edema observed, the distribution of the fluid in this case being general and the amount extreme.

The most constant feature of the nephritic cases was the early onset of an acute confusional mental state, progressing rapidly to noisy and active delirium, with incontinence of urine and feces, and every appearence of profound toxemia.

The volume of urine in each 24 hours was seldom diminished more than might have been expected with the high fever and pneumonia, the lowest average daily output for the first two days being 30 oz. per 24 hours, the highest 65 oz.

Microscopical examination of the centrifuged urine showed an abundance of casts, both granular and hyaline, with a striking absence of blood cells.

The postmortem examinations showed in most cases an enlarged and firm spleen. Microscopical examination of the kidneys showed in at least four cases (possibly also in four others) evidence of pre-existing renal disease.

The present observations seem to show that persons whose kidneys are previously damaged by disease are especially prone to die from bronchopneumonia in this epidemic.

